# Mutualistic Coupling Between Vocabulary and Reasoning in Young Children: A Replication and Extension of the Study by Kievit et al. (2017)

**DOI:** 10.1177/0956797619841265

**Published:** 2019-05-17

**Authors:** Rogier A. Kievit, Abe D. Hofman, Kate Nation

**Affiliations:** 1 MRC Cognition and Brain Sciences Unit, University of Cambridge; 2 Max Planck UCL Centre for Computational Psychiatry and Ageing Research, University College London; 3 Department of Psychological Methods, University of Amsterdam; 4 Department of Experimental Psychology, University of Oxford

**Keywords:** intelligence, development, reasoning, vocabulary, psychometrics, open data

## Abstract

Recent work suggests that the positive manifold of individual differences may arise, or be amplified, by a mechanism called *mutualism*. Kievit et al. (2017) showed that a latent change score implementation of the mutualism model outperformed alternative models, demonstrating positive reciprocal interactions between vocabulary and reasoning during development. Here, we replicated these findings in a cohort of children (*N* = 227, 6–8 years old) and expanded the findings in three directions. First, a third wave of data was included, and the findings were robust to alternative model specifications. Second, a simulation demonstrated that data sets of similar magnitude and distributional properties could have, in principle, favored alternative models with close to 100% power. Third, we found support for the hypothesis that mutualistic-coupling effects are stronger and self-feedback parameters weaker in younger children. Together, these findings replicated the work of Kievit et al. (2017) and further support the hypothesis that mutualism supports cognitive development.

A canonical finding in psychology is the *positive manifold*: the observation that individual differences in cognitive abilities are universally positively correlated. The traditional explanation of this finding is to posit that a dominant underlying ability affects task performance across domains—a so-called *g* factor. However, new mathematical models instead propose alternative-generating mechanisms that predict identical cross-sectional data (Van Der Maas et al., 2006, p. 843). One such model, the so-called *mutualism* model (Van Der Maas et al., 2006), demonstrates how the positive manifold may arise from positive, reciprocal developmental interactions between cognitive abilities. In line with this hypothesis, previous work suggests that facilitatory interactions likely play a role in cognitive development across the life span (e.g., Ferrer & McArdle, 2004; McArdle, Hamagami, Meredith, & Bradway, 2000). Using latent change score models, Kievit et al. (2017) showed evidence in favor of the mutualism model over two alternative accounts—investment theory and developmental *g*-factor theory—in a sample of adolescents (ages 14–25 years) in the domains of vocabulary and reasoning.

Here, we report a replication and extension of Kievit et al.’s (2017) findings in an independent data set of younger children, ages 6 to 8 years. We used the same measures and model-comparison strategies and expanded on the original findings by incorporating a third wave of testing, a simulation, and robustness analyses and by testing (and supporting) the prediction that mutualistic-coupling effects would be stronger in younger children.

## Method

A sample of 227 individuals (129 girls) took part in four waves of testing, at ages 4.8, 6.22, 7.22, and 8.22 years. At each wave, vocabulary and matrix reasoning were measured using the Wechsler Abbreviated Scale of Intelligence (Wechsler, 2011). The study received ethical approval from the University of Oxford Ethics Committee. Because Wave 1 used the Wechsler Preschool and Primary Scale of Intelligence instead of the Wechsler Abbreviated Scale of Intelligence, we analyzed data from Waves 2 to 4 only. Raw scores and descriptive statistics are shown in Table S1 in the Supplemental Material available online; raw scores are plotted as a function of age in Figure S1 in the Supplemental Material. The raw data and analysis code are freely available on the Open Science Framework (OSF) at https://osf.io/xf7rn/. Simulations (see below and code on the OSF) suggest that although the current cohort is a convenience sample, the sample size is more than sufficient for adequate parameter recovery and model comparison.

**Fig. 1.**
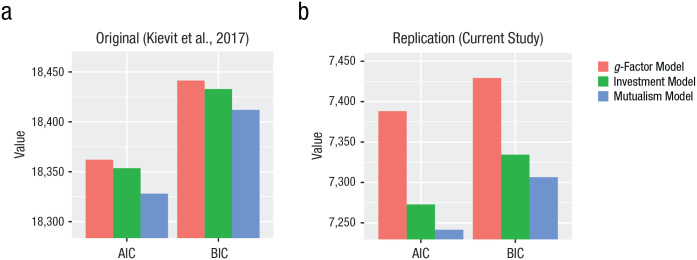
Akaike information criterion (AIC) and Bayesian information criterion (BIC) for the three models in the original article (a) and in the current replication (b). Lower values reflect better fit.

We used the same approach as Kievit et al. (2017), comparing a series of (bivariate) latent change score models (sometimes referred to as latent difference score models; e.g., McArdle & Hamagami, 2001) to capture cognitive development across three waves. As before, we compared three different theoretical accounts: *g*-factor theory, investment theory, and mutualism (see Kievit et al., Fig. 1). All models were fitted using *lavaan* (Rosseel, 2012) in the R programming environment (R Core Team, 2016) using robust maximum-likelihood estimation with Yuan-Bentler correction for deviations from multivariate normality and full-information maximum likelihood to account for missing data. Although the analyses were not preregistered (because cohort data collection was already complete), the data set was shared (July 2017) with the lead author only after acceptance of the original manuscript (April 2017, including public deposition of the analysis code); the code in the original manuscript as well as the replication are publicly available to demonstrate the similarities. As in the study by Kievit et al., no data were removed prior to the analysis. We first present a replication of the precise model comparisons from Kievit et al.’s study using the same code, but expanded to accommodate the third wave of data, as well as an alternative implementation of the mutualism model as a parallel-process model. We then present results from tests of the prediction that mutualistic-coupling effects are stronger in younger children.

## Results

We used the same analysis scripts as in the original analyses (https://osf.io/rvcph/files/) to replicate Kievit et al.’s (2017) findings. Because the new data included three waves of data rather than two, we imposed default equality constraints on the same parameter across waves whenever tenable but estimated parameters freely when necessary. We made one improvement to the previous model specification on the basis of psychometric considerations, namely, integrating a latent layer for the observed scores. Because single-indicator latent variables can pose challenges to model estimation (cf. Newsom, 2015), we accounted for measurement reliability by imposing residual variances on the observed scores according to the formula proposed by Brown (2006, p. 139) and others, namely, *S*^2^(1 – *Rxx*), where *Rxx* is the (manual-derived) internal consistency estimate, and *S*^2^, the sample variance of each variable (we note that omitting this adjustment yields virtually identical results and model comparisons). As before, we imposed measurement invariance for the *g*-factor model across occasions. As can be seen in Figure 1, model comparison shows strikingly similar results to those reported by Kievit et al. The *g*-factor model fitted most poorly, followed by the investment model. The mutualism model showed the best fit, χ^2^(8) = 9.849, *p* = .276, root-mean-square error of approximation (RMSEA) = 0.032, 90% confidence interval (CI) = [0.00, 0.088], comparative fit index (CFI) = .996, standardized root-mean-square residual (SRMR) = .032. Details of model fit are shown in Table S2 in the Supplemental Material.

A likelihood-ratio test of the investment-theory model versus the mutualism model also replicated the original findings, favoring the mutualism model, Δχ^2^(1) = 36.207, *p* < .001. Because the mutualism model is also the most complex model, we used information indices (Akaike information criterion, or AIC, and Bayesian information criterion, or BIC) to give a parsimony-weighted model comparison, as shown in Figure 1. Despite being the most complex model, the mutualism model considerably outperformed the other two candidates, as in the study by Kievit et al. (2017). As before, a multigroup model with gender as the grouping factor provided no evidence for differences, Δχ^2^(13) = 18.338, *p* = .1451. In the investment model as well as the mutualism model, we freed the vocabulary-change intercept across Waves 2 and 3 because it significantly improved overall model fit (conditional vocabulary improvement was greater between Waves 1 and 2 than between Waves 2 and 3); fully constraining to equality across all models did not meaningfully affect key parameters or model comparisons. We then examined the parameter estimates in more detail.

**Fig. 2.**
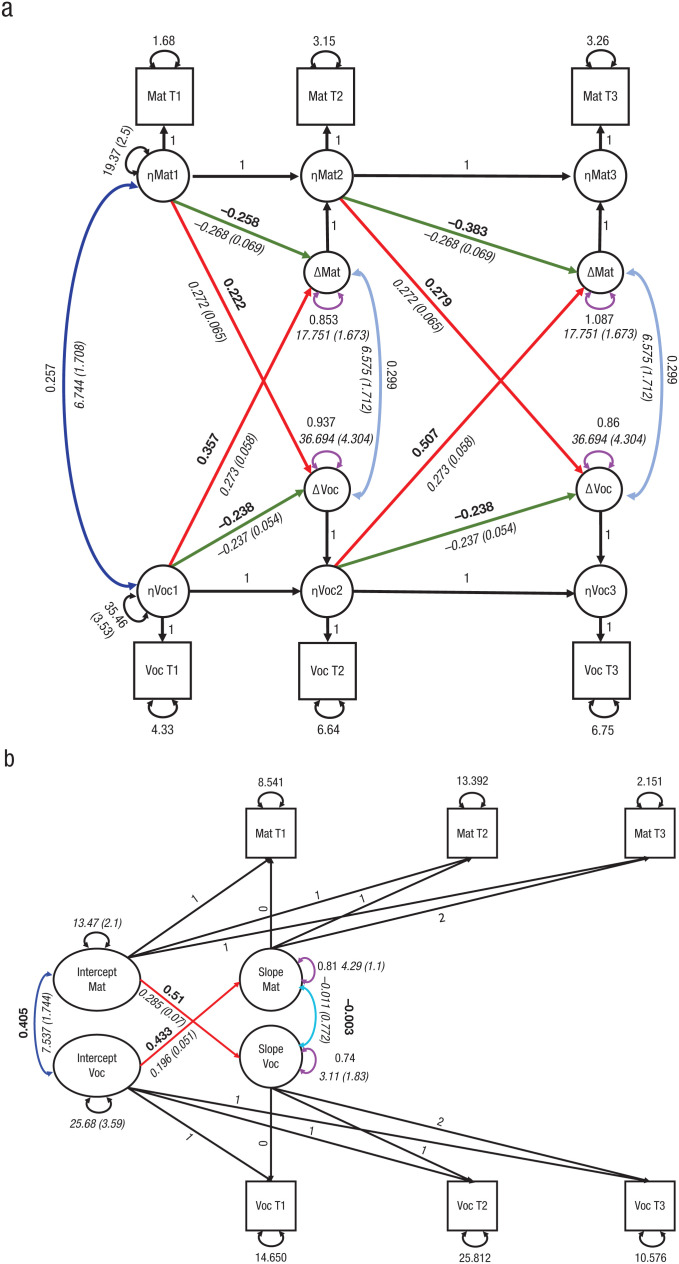
Estimated parameters for the mutualism model implemented as a latent change score model (a) or a parallel-process model (b). Values in roman are standardized parameter estimates, and values in italics are unstandardized parameter estimates (with standard errors in parentheses). Key standardized parameters of interest are highlighted in boldface. Paths between key parameters of interest are highlighted in color for purposes of readability. The linear slope is captured by constraining the factor loadings of the slope factor for successive waves to 0, 1, and 2. For the latent change score model, equality constraints are imposed on the same parameter across waves as the default. The only exception is the conditional intercept for the vocabulary-change scores. Intercepts are estimated but not shown for visual clarity. Latent change scores were allowed to freely correlate over time (not shown for visual clarity). Voc = vocabulary; Mat = matrix reasoning; T1 = Time 1; T2 = Time 2, T3 = Time 3.

The final mutualism model is shown in Figure 2a, including all standardized and raw parameter estimates (with standard errors for the latter). Next, we examined an explicit prediction made by Kievit et al. (2017) prior to receiving the current data set. The original sample comprised (late) adolescents, ages 14 to 25 years, whereas the current sample was much younger (6–8 years). Because cognitive development is more rapid in younger children than in adolescents and therefore more likely to be malleable, Kievit et al. predicted that “the coupling effects we observed are likely to be stronger earlier in life and the self-feedback parameters weaker, as developmental change in higher cognitive abilities is most rapid during pre- and early adolescence” (p. 1428).

Inspection of the final model fit, as shown in Figure 2, reveals that both of these predictions are in line with the data. The negative self-feedback parameters (green arrows) were considerably weaker in this sample of children, likely because the younger children were not as near their developmental asymptote. In the original adolescent sample (Neuroscience in Psychiatry Network, or NSPN), the standardized self-feedback parameter (β) for reasoning was −0.602, whereas in the younger Oxford sample, it was −0.27 and −0.39 for Waves 2 and 3, respectively (Steiger’s *z*s = 5.7 and 3.86, respectively). Similarly, the vocabulary self-feedback path had a β of −0.362 in the adolescent NSPN sample but was −0.189 and −0.227 in the Oxford sample for Waves 2 and 3, respectively (*z*s = 2.45 and 1.93, respectively).

Next, we examined the more crucial prediction: that of mutualistic coupling, predicted to be stronger in younger children. Indeed, in the NSPN sample, the coupling parameter (*r*) from reasoning to changes in vocabulary was .155 (standardized estimate), whereas in the younger Oxford sample, it was .218 and .276 for Waves 2 and 3, respectively (red arrows pointing downward in Fig. 2; *z*s = 0.92 and 1.72, respectively). Similarly, the positive coupling from vocabulary to reasoning development had a β of 0.203 in the adolescent NSPN sample but was 0.329 and 0.449 in the Oxford sample for Waves 2 and 3, respectively (red arrows pointing upward in Fig. 2; *z*s = 2.21 and 4.66, respectively). This demonstrates that children with higher vocabulary scores made larger gains in reasoning ability, and children with lower vocabulary scores made smaller gains in reasoning ability. These results show that all parameter comparisons are in the predicted direction, and most are statistically significantly different. Moreover, these findings replicated the relative dominance of vocabulary in driving reasoning development.

Finally, the residual correlation between contemporaneous change scores was both positive and stronger (.299 vs. .1003, *z* = 2.74) in younger children than in the adolescents, suggesting that whatever additional factors may drive change in both domains do so more strongly in younger children. The dynamic coupling in the Oxford sample is shown in Figure S2 in the Supplemental Material, which illustrates the stronger mutualistic effect using a vector plot. Taken together, these findings show that, as before, the mutualism model considerably outperformed both the *g*-factor model and the investment-theory model in explaining development between two domains and that these effects are stronger in young children.

### Alternative model specification

The material above illustrates how we tested the mutualism hypothesis using a three-wave latent change model on the observed scores. To examine the robustness of our conclusions to alternate specifications, we reestimated the model as a *parallel-process model* (see Fig. 2). This is a type of bivariate latent growth model that estimates slopes and intercepts as in typical growth models but regresses each intercept on the slope of the other domain. This allows one to estimate the driving force of the baseline in one domain on the rate of change in the other. This provides an intuitive implementation of the mutualism model: Mutualism would predict that the intercept of one domain would drive the rate of gain in the other (e.g., vocabulary to reasoning, and vice versa). This model showed adequate fit to the data, χ^2^(9) = 18.72, *p* = .028, RMSEA = 0.069, 90% CI = [0.022, 0.113], CFI = .978, SRMR = .048. Crucially, the core prediction of the mutualism model was further validated: The path of vocabulary intercepts on reasoning slopes was strong and positive (b = 0.196, *SE* = 0.051, β = 0.43), and constraining this path led to a considerable drop in model fit, χ^2^(1) = 18.72, *p* < .0001. The path from reasoning intercepts on vocabulary slope was similarly strong and positive (b = 0.285, *SE* = 0.070, β = 0.51), and constraining it led to a comparable drop in model fit, χ^2^(1) = 12.64, *p* < .001. Moreover, both standardized paths were significantly stronger than in the older NSPN sample (*z*s = 4.71 and 4.2, respectively), although they were less easy to compare given different model specifications. In contrast, freeing the within-domain driving effects (e.g., vocabulary intercepts driving vocabulary slopes, and reasoning intercepts driving reasoning slopes) led to a worsening of model fit (ΔAIC = 3.6, ΔBIC = 10.4), χ^2^(2) = 0.42, *p* = .81 (in favor of the model with only cross-domain coupling). This favored cross-domain coupling in line with mutualism, rather than a within-domain “Matthew-effect” account (the effect that a higher starting point in a given domain leads to greater gains in that same domain—the “rich get richer”; for an explanation within reading, see Stanovich, 2009).

Finally, we fitted a three-wave random-intercept cross-lagged panel model (Hamaker, Kuiper, & Grasman, 2015), which yielded similar conclusions, including positive bidirectional coupling, demonstrating that our interpretation of the mutualism effect in this data set was robust to alternate model specifications. Together, and in line with a priori predictions (Kievit et al., 2011, p. 154; Van Der Maas et al., 2006, p. 844), these findings showed that longitudinal interactions between cognitive domains are facilitatory (positive) and that they may help explain the positive manifold. Depending on the time and variable resolution of the data, other implementations and extensions of the mutualism model as psychometric models are possible, such as the autoregressive-latent-trajectory model, the inclusion of structured residuals or time-varying covariates, mixture models (that may tease apart subpopulations that differ as a function of key parameters), and continuous-time models (that treat time as a continuous factor; McArdle, 2009; Newsom, 2015), or an entirely different class of models known as network models (Van Der Maas, Kan, Marsman, & Stevenson, 2017).

### Simulation

It is important to ensure that our model specification does, in principle, allow for the *g*-factor latent change score model to be the best model in a sample of this magnitude and structure (two variables, three waves; *N* = 227). To do so, we simulated data (see https://osf.io/xf7rn/) under a hypothesized plausible *g*-factor latent change model most similar in spirit to a mutualism model: one in which there are steady increments in the latent *g* factor and modest positive self-feedback. In other words, individuals with higher *g* will, on average, make slightly greater improvements over time than individuals with lower *g*. We simulated data for three waves (*N* = 227, as above) using this model (code available at https://osf.io/xf7rn/) and ensured that raw data as well as the covariance matrix closely resembled our observed data. Next, we fitted the true *g*-factor model to these simulated data, which showed excellent model fit and good parameter recovery. Even with a simulated sample size as low as 50, model convergence, model fit, and parameter recovery remained good. Thus, there was no a priori reason that the *g*-factor model of change could not have fit our data well.

Simulating data in this manner also allowed us to ask about power and model selection: If we fitted both the *g*-factor latent change score model and the more complex mutualism model to data generated under the *g*-factor model, how often did we (rightly) prefer the *g*-factor model? That is, is there a risk that the mutualism model overfits to data truly generated under the *g*-factor model? To examine this question, we simulated 1,000 data sets for a total sample of 227 under the *g*-factor latent change score model specified above and used the AIC for model comparison (because the models were not nested). In 999 of 1,000 iterations, the *g* factor was preferred, suggesting 99.9% power to prefer the *g*-factor model if it was indeed the data-generating mechanism. Similar results were obtained when we changed the self-feedback parameter for the *g*-factor model to 0 or to −0.2 (and increased η_*g*_ to ensure global improvement over time). Together, these simulations conclusively showed that the *g*-factor model can, in principle, be a good model of developmental change in cognitive abilities, but it is not an accurate empirical reflection of our data set.

Finally, we investigated statistical power within the latent change score model; given our sample size and model specification, what is our statistical power to detect a reliable parameter estimate for the key (coupling) parameters? We simulated 100 samples for a range of known coupling-parameter strengths and calculated the proportion of simulations for which the coupling parameter was nominally statistically significant at an alpha of .05 (see Fig. S3 in the Supplemental Material). With our current model specification and sample size, we had 73% power to detect a small coupling effect (*r* = .1, as defined by Gignac & Szodorai, 2016) but effectively 100% power to detect intermediate (*r* = .2) and large (*r* = .3) effects.

## Discussion

Here, we replicated and extended the findings reported by Kievit et al. (2017), showing striking similarities in model comparison across two independent samples. Notably, the new data were in line with the prediction made by Kievit et al. that mutualistic-coupling effects would be stronger (and self-feedback effects weaker) in younger children than in adolescents.

Although the empirical observations here offer clear support for mutualism, the precise mechanisms that support mutualism-type effects remain poorly understood. Previously, researchers have proposed several mechanisms (e.g., Van Der Maas et al., 2006, p. 845), including semantic bootstrapping. It may be that children with better vocabulary and verbal skills are more able to efficiently decompose abstract problems into constituent “rules.” The availability of verbal resources may also lower demands on working memory for maintaining and applying such rules. For instance, Gathercole, Service, Hitch, Adams, and Martin (1999) found a close association between vocabulary and phonological short-term memory, in line with the notion that greater vocabulary skills may facilitate the working memory demands of matrix rule decomposition.

A second, complementary class of mechanisms is *environmental*, namely, that processes or features of the environment facilitate mutualistic development (in line with the environmental-multiplier hypothesis posited by Dickens & Flynn, 2001). For instance, high ability in one cognitive domain may induce (e.g., through selection) a more challenging opportunity that facilitates broader skill development, much like relative age effects consistently observed in various types of sport (e.g., Hancock, Adler, & Côté, 2013). Direct empirical testing of the environmental multiplier (e.g., Van Der Maas et al., 2017, Fig. 2) will require detailed, ideally longitudinal, data on the quality of the learning environment. We hypothesize that in a sufficiently large data set, coupling parameters are stronger in more advantageous learning environments.

This study had limitations of generalization similar to those in our previous report. Although here we tested our models across three developmental points rather than two, we still focused on only the two domains of vocabulary and reasoning, each measured by a single indicator; “latent” in our latent change score model refers purely to the change scores (cf. McArdle, 2009, Fig. 2b). A more fully latent specification, including a measurement model at every occasion, would be preferable (e.g., Kievit et al., 2018, Fig. 3), to better incorporate measurement error and establish measurement invariance over time. Encouragingly, however, Hofman et al. (2018) reported a replication in the domain of arithmetic, showing mutualistic coupling between latent estimates of addition and counting, as well as multiplication and division. Although the support for the mutualism model here is considerable, other phenomena traditionally associated with the mutualism model, such as increasing correlations across development, are sometimes (e.g., Hofman et al., 2018) but not always (e.g., Gignac, 2014) observed. Moreover, positive cognitive coupling has been reported among many, but not necessarily all, possible pathways (e.g., see Ferrer & McArdle, 2004; McArdle, Ferrer-Caja, Hamagami, & Woodcock, 2002). However, uniform positive coupling is not required by mutualism—merely that the preponderance of pathways is positive. Taken together, these findings suggest, as in the study by Kievit et al. (2017), that “a model of intellectual development that omits coupling parameters is incomplete” (p. 1427). Together, these findings provide further support for mutualistic processes as essential for a more complete understanding of cognitive development in childhood and adolescence. Future examination in large, longitudinal samples with standardized cognitive tests (e.g., Adolescent Brain Cognitive Development study; Volkow et al., 2018) and complementary (neural, mental health) measures will help further elucidate how the dynamic interplay between domains may support cognitive development.

## Supplemental Material

KievitOpenPracticesDisclosure_rev – Supplemental material for Mutualistic Coupling Between Vocabulary and Reasoning in Young Children: A Replication and Extension of the Study by Kievit et al. (2017) Supplemental material, KievitOpenPracticesDisclosure_rev for Mutualistic Coupling Between Vocabulary and Reasoning in Young Children: A Replication and Extension of the Study by Kievit et al. (2017) by Rogier A. Kievit, Abe Hofman and Kate Nation in Psychological Science

KievitSupplementalMaterial_rev – Supplemental material for Mutualistic Coupling Between Vocabulary and Reasoning in Young Children: A Replication and Extension of the Study by Kievit et al. (2017) Supplemental material, KievitSupplementalMaterial_rev for Mutualistic Coupling Between Vocabulary and Reasoning in Young Children: A Replication and Extension of the Study by Kievit et al. (2017) by Rogier A. Kievit, Abe Hofman and Kate Nation in Psychological Science
