# Supplementary material for: Mutualistic Coupling Between Vocabulary and Reasoning in Young Children: A Replication and Extension of the Study by Kievit et al. (2017)
Source: Psychol Sci. 2019 May 17;30(8):1245–52. doi: 10.1177/0956797619841265 (PMC6691592; doi:10.1177/0956797619841265)
Supplement: KievitSupplementalMaterial_rev – Supplemental material for Mutualistic Coupling Between Vocabulary and Reasoning in Young Children: A Replication and Extension of the Study by Kievit et al. (2017) [file KievitSupplementalMaterial_rev.pdf]

Supplementary figures and tables

Figure S1: Raw data across three waves

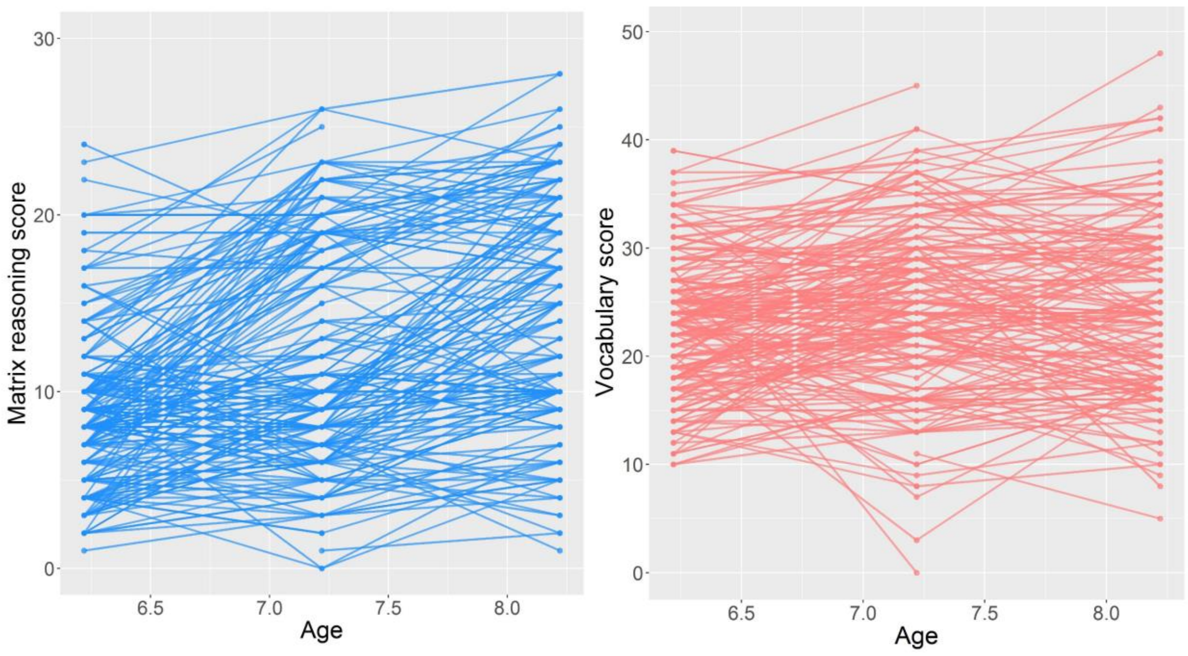

Figure S2: Vector plot of model-implied changes for the latent change score specification. On the left, we show the mutualism effect in adolescents (highlighted as the size of the blue and red panels, which reflects the asymmetry in predicted cognitive change). On the right, we show the much stronger effect in the Oxford sample – not only is the aggregate change greater, the dependency on cross-domain coupling is visibly stronger.

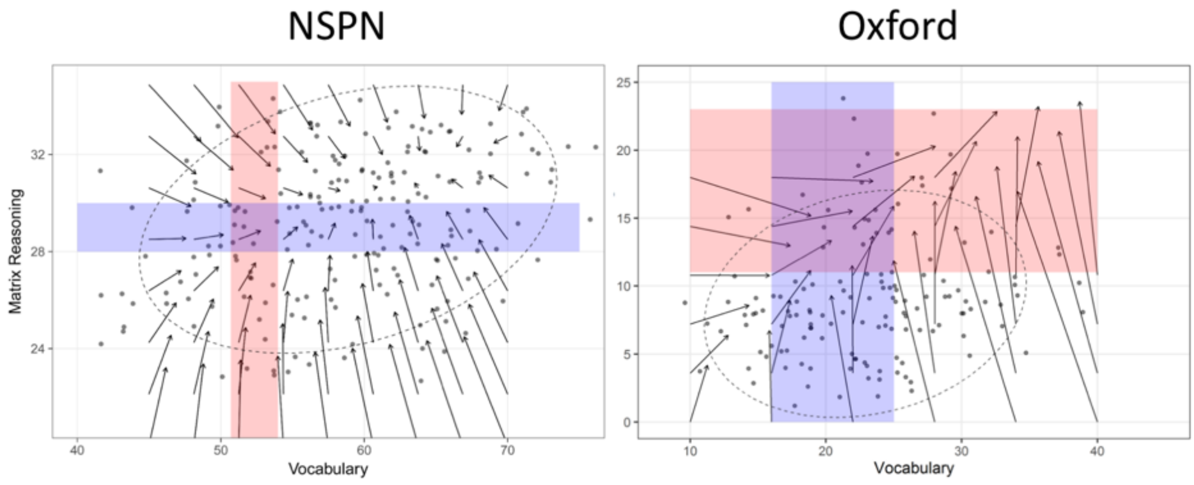

Figure S3: Power analysis. Bars reflect raw/standardized betas for the simulated coupling strength.

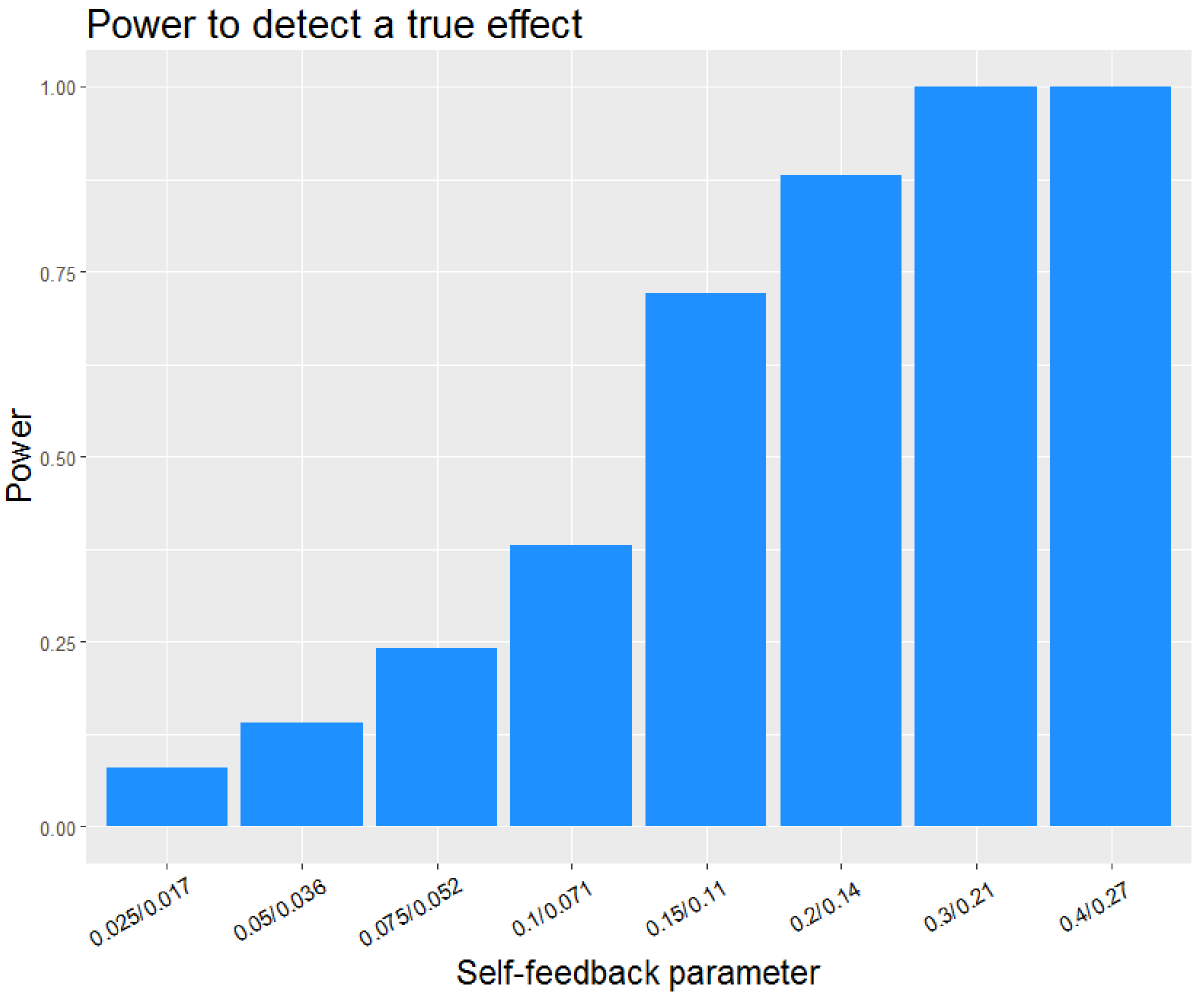

Table S1: Raw Scores and Descriptive Statistics for Matrix Reasoning and Vocabulary Scores across three waves.

Task	N	Min	Max	Mean	SD	Skewness	Kurtosis
Matrix reasoning T1	215	1	24	8.86	4.58	0.98	0.96
Matrix reasoning T2	202	0	26	12.05	6.27	0.33	-0.97
Matrix reasoning T3	172	1	28	14.53	6.38	-0.03	-1.04
Vocabulary T1	213	10	39	22.77	6.27	0.16	-0.41
Vocabulary T2	202	0	45	24.57	7.77	-0.24	0.00
Vocabulary T3	172	5	48	24.20	7.84	0.29	-0.29

Table S2: Fit Statistics for Each of the Three Models

	χ2	df	p(χ2)	RMSEA	CFI	SRMR
g factor	165.42	15	<0.0001	0.21 [0.182 - 0.239]	0,654	0,161
Investment	43,311	9	<0.0001	0.13 [0.092 - 0.170]	0,921	0,092
Mutualism	9,849	8	0.27	0.032 [0.000 - 0.088]	0,992	0,027
